# Improved supercapacitor performances by adding carbonized C_60_-based nanospheres to PVA/TEMPO-cellulose hydrogel-based electrolyte†

**DOI:** 10.1039/d3ra03349j

**Published:** 2023-07-18

**Authors:** Han Jia, Sabina Shahi, Lok Kumar Shrestha, Katsuhiko Ariga, Tsuyoshi Michinobu

**Affiliations:** a Department of Materials Science and Engineering, Tokyo Institute of Technology 2-12-1 Ookayama Meguro-ku Tokyo 152-8552 Japan michinobu.t.aa@m.titech.ac.jp; b Central Department of Chemistry, Tribhuvan University Kirtipur Kathmandu 44613 Nepal; c Research Center for Materials Nanoarchitectonics (MANA), National Institute for Materials Science (NIMS) 1-1 Namiki Tsukuba 305-0044 Japan SHRESTHA.Lokkumar@nims.go.jp; d Department of Materials Science, Faculty of Pure and Applied Sciences, University of Tsukuba 1-1-1 Tennodai Tsukuba 305-8573 Japan; e Department of Advanced Materials Science, Graduate School of Frontier Sciences, The University of Tokyo 5-1-5 Kashiwanoha Kashiwa 277-8561 Japan

## Abstract

With the emergence of the energy crisis and the development of flexible electronics, there is an urgent need to develop new reliable energy supply devices with good flexibility, stable energy storage, and efficient energy transfer. Porous carbon materials have been proven to enhance the efficiency of ion transport, as the nanospaces within them serve as pathways for mass transport. However, they have been mainly investigated in the electrodes of supercapacitors and batteries. To elucidate their function in the solid electrolytes, we introduced C_60_-based carbonized nanospheres into PVA/TEMPO-cellulose-based hydrogels by exploiting the electrostatic interaction between the carboxyl groups of TEMPO-cellulose and the carbonized nanospheres. The obtained hydrogels were further utilized as the solid electrolytes for the supercapacitors. Through a comprehensive investigation, we found that the carbonized nanospheres can act as physical crosslinking points and increase the maximum stress of the hydrogel from 0.12 to 0.31 MPa without affecting the maximum strain. In addition, the nanospaces of the carbonized nanospheres provided a pathway for ion transport, improving the capacitance of the supercapacitor from 344.83 to 369.18 mF cm^−2^ at 0.5 mA cm^−2^. The capacitance retention was also improved from 53% to 62% at 10 mA cm^−2^. Collectively, this study provides new insights into the application of carbonized materials to solid electrolytes.

## Abstract 2

Carbonized C_60_ nanospheres efficiently improve the mechanical properties and supercapacitor performance when they are added to a poly(vinyl alcohol)/TEMPO-cellulose hydrogel-based electrolyte.

## Graphical abstract

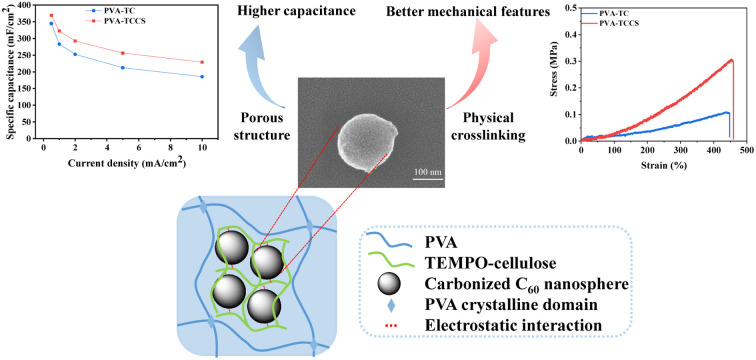

## Introduction

Recently, the rapid development of flexible electronics has attracted a great amount of attention. However, most conventional energy supplier devices lack flexibility.^1^ To address the incompatibility between flexible electronics and rigid power sources, the next generation of flexible power supplies must be developed. Supercapacitors, one of the most promising electrochemical energy storage systems, have great potential in the field of flexible power supplies due to their high power density, ultra-fast charging rate, low-cost production, and simple device structure.^2–5^ Unlike batteries, supercapacitors are electrochemical double-layer capacitor devices that store energy through electrostatic interactions rather than chemical reactions. Therefore, supercapacitors can stably store and quickly release energy during charge and discharge cycles.^6–8^ Flexible supercapacitors always require a solid electrolyte with good mechanical properties, stable electrolyte storage capacity, and firm contact with the electrode to avoid damage due to the bending or stretching of the devices during operation.^9–17^ Hydrogels are crosslinked polymer networks swollen in aqueous solutions. Because of the large electrolyte capacity, flexibility, and lack of leakage, hydrogels are considered as potential flexible electrolytes and have attracted more and more attention. For example, Yao *et al.* recently fabricated a flexible zinc-ion hybrid supercapacitor using a cellulose hydrogel electrolyte swollen with a high concentration of ZnCl_2_.^18^ By incorporating the hydrogel in a Zn anode and an activated carbon cathode (AC), the fabricated supercapacitor showed an outstanding performance (193 mA h g^−1^ capacity, 192 W h kg^−1^ energy density, and 16 976 W kg^−1^ power density). In addition, the anti-freezing property of the concentrated ZnCl_2_ electrolyte ensured stable operation even at −20 °C. Li and co-workers reported a conductive zwitterionic hydrogel electrolyte synthesized *via* copolymerization of acrylamide and zwitterionic monomer in an H_2_SO_4_/ethylene glycol aqueous solution.^9^ The assembled supercapacitor with activated carbon/carbon black (AC/CB) displayed a high specific capacitance of 93.5 F g^−1^.

As in the above examples, the reported hydrogel electrolyte-based supercapacitors have shown outstanding performances, but the high cost, complicated synthesis, and use of concentrated electrolyte solutions are inevitable in the fabrication process. We now report a hydrogel electrolyte with a simple structure by introducing porous hollow carbon nanospheres into the polymer network of the hydrogel.

Porous carbon-based materials, especially porous carbon nanospheres, have been widely studied as electrodes for energy storage devices, such as supercapacitors and lithium-ion batteries, because they are easy and inexpensive to prepare and can facilitate the transport of various chemical species.^19–21^ Among various properties of the porous carbon nanospheres, the uniform size is one of the most important factors in supercapacitor performances. Smaller particle sizes and higher monodispersity of carbon nanospheres always lead to better performances because of lower mass transport and charge transfer resistances.^21–23^

Fullerene C_60_ is a well-known zero-dimensional molecule with the ability to self-assemble to form various morphologies, including nanospheres.^24–27^ The rich π-electrons, controllable morphology, and high specific surface area of C_60_-based materials make them applicable as nanoporous carbon electrode materials. Many reports have revealed the great potential of C_60_-based electrodes in energy storage devices.^28,29^ However, little research has been done on the effects of porous carbon nanospheres on solid electrolytes. Notably, because of the much larger size, the C_60_-based carbonized spheres (CS) cannot be as easily and uniformly dispersed in the hydrogel matrix as carbon dots.^30^ Therefore, in this work, CS was introduced into poly(vinyl alcohol) (PVA)/2,2,6,6-tetramethylpiperidine-1-oxyl radical-oxidized cellulose (TEMPO-cellulose, TC)-based hydrogels *via* electrostatic interactions with the carboxylate groups of the TEMPO-cellulose. The obtained hydrogels showed improved mechanical properties due to the deformability of CS, which could effectively suppress the stress concentration and crack growth.^31^ The performance of the assembled supercapacitor was improved due to the enhanced ionic diffusion of electrolytes through the nanospace.

## Experimental section

### Material

Fullerene C_60_ (pC_60_: 99.5%) was purchased from BBS Chemicals (Texas, USA). Isopropyl alcohol (IPA, 99.7%), *m*-xylene (99.8%), and ethylenediamine (EDA, 99%) were purchased from Wako Chemicals Corporation (Tokyo, Japan). 2,2,6,6-Tetramethylpiperidine-1-oxyl radical-oxidized cellulose (TEMPO-cellulose, TC) was supplied from Nippon Paper Industries Co., Ltd. (Tokyo, Japan). Poly(vinyl alcohol) (PVA, *M*_w_ 146 000–186 000), carbon black (CB), activated carbon (AC), and poly(vinylidene fluoride) (PVDF, *M*_w_ 534 000) were obtained from Sigma-Aldrich (Tokyo, Japan). *N*-Methyl-2-pyrrolidone (NMP, 99.0%) was procured from Kanto Chemical Co., Inc (Tokyo, Japan). All chemicals were used as received.

### Preparation of C_60_-based carbonized spheres (CS)

The C_60_-based nanospheres were synthesized as previously reported.^32^ A solution of EDA (0.55 mL) in *m*-xylene (5 mL) was prepared by applying sonication for 30 min. 5 mL of a C_60_ solution in *m*-xylene (1 mg mL^−1^) was then added to the EDA/*m*-xylene solution, and the obtained solution was thoroughly mixed on a vortex mixer for 10 s. After 40 min of incubation, the precipitates in the solution were collected by centrifugation at 9500 rpm for 10 min and repeatedly washed with IPA and DI-water 3 times, respectively. The resultant precipitates were dispersed in water/IPA (volume ratio = 5 : 1) and freeze-dried for 24 h to yield C_60_ nanospheres. The C_60_ nanospheres were then carbonized at 1100 °C in a tube furnace (KOYO, Tokyo, Japan) under an inert nitrogen gas atmosphere to obtain the hierarchically porous hollow carbonized spheres (CS). The heating ramp, hold time, and nitrogen gas flow rate was 10 °C min^−1^, 3 h, and 120 cc min^−1^, respectively, during the carbonization process.

### Preparation of PVA/TEMPO-cellulose/CS hydrogel (PVA-TCCS)

The obtained CS was added to a TC aqueous solution (1 wt%) and stirred for 12 h followed by sonication for 1 h, yielding a well-dispersed solution with 1 wt% of CS. 10% of PVA was then dissolved in the TCCS solution at 95 °C with constant stirring for 1 h. The resultant solution was centrifugated at 2000 rpm for 1 min to remove bubbles. To obtain hydrogels with good mechanical properties, the mixed solution was then frozen at −20 °C for 12 h and thawed twice.

### Preparation of hydrogel-based supercapacitor

The hydrogel electrolyte was prepared by immersing the hydrogel in an H_2_SO_4_ aqueous solution (1 M) for 12 h. Every 3 h, the H_2_SO_4_ aqueous solution was replaced with a new H_2_SO_4_ aqueous solution to ensure that the water was completely replaced. The resultant hydrogel electrolyte was cut into squares about 1 cm long, 0.1 cm thick, and 0.6 g in weight to perfectly fit on the electrode surface. The working electrodes were fabricated as follows. The activated carbon (AC), conductive carbon black (CB), and poly(vinylidene fluoride) (PVDF) with a weight ratio of 8 : 1 : 1 were ground and dispersed in an appropriate amount of NMP. The dispersion was then uniformly applied on a 1 cm^2^ area of carbon paper and placed in a vacuum oven at 80 °C overnight to obtain the electrodes. The loading mass of active materials was around 5 mg on each electrode. A solid-state supercapacitor with a “sandwich structure” was fabricated by covering both sides of a hydrogel electrolyte with two electrodes of the same area (1 cm^2^) loaded with active materials. The supercapacitor was then encapsulated using a VHB tape (3 M) to maintain the integrity and prevent water absorption.

### Characterization

The chemical structures of the hydrogels were investigated by Fourier transform infrared (FTIR) spectroscopy (FT/IR-4200, JASCO, Tokyo, Japan) from 4000 to 500 cm^−1^ at 20 °C. The morphology of the CS was examined using scanning electron microscopy (SEM) (S-4800, Hitachi Co., Ltd. Tokyo, Japan) operated at 10 kV and 10 μA. The hydrogel structures were observed using SEM (JEM-7500F, JEOL, Tokyo, Japan). The hydrogel samples were freeze-dried before the observation. Thermogravimetric analysis (TGA) was carried out on an STA 2500 Regulus Simultaneous Thermal Analysis (NETZSCH, SELB, Germany). The differential scanning calorimetry (DSC, DSC8230, Thermo Plus EVO, Tokyo, Japan) tests were conducted at a scanning rate of 10 °C min^−1^ from −80 to 20 °C under a nitrogen flow. The crystallization structures of the hydrogel samples were characterized by X-ray diffraction (XRD, Mini Flex 600, Rigaku, Tokyo, Japan). The strain–stress curves of the hydrogels were measured using a testing machine (Toyoseiki Strograph-VES5D, Tokyo, Japan) at a rate of 10 mm min^−1^.

The electrochemical performance of the as-fabricated supercapacitors was measured by cyclic voltammetry (CV), galvanostatic charge/discharge (GCD), and electrochemical impedance spectroscopy (EIS). The electrode-specific capacitance (*C*_A_, mF cm^−2^), energy density (*E*, μWh cm^−2^), and power density (*P*, μW cm^−2^) of the supercapacitor are calculated according to the following equations,^11^1
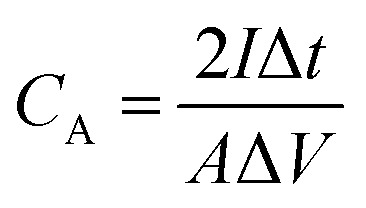
2
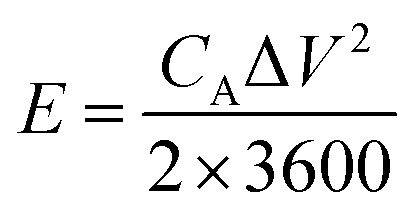
3
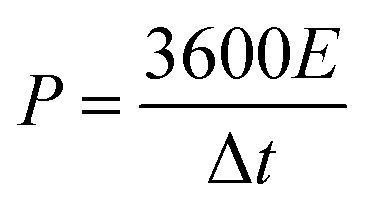
where *C*_A_ is the areal specific capacitance, *I* is the constant discharge current (mA), Δ*t* is the discharge time (s), *A* is the area (cm^2^) of an electrode, Δ*V* (V) is the operating voltage excluding potential drop, *E* is the volumetric energy density, and *P* is the volumetric power density.

## Results and discussion

### Controlling C_60_-based nanospheres

**Fig. 1:**
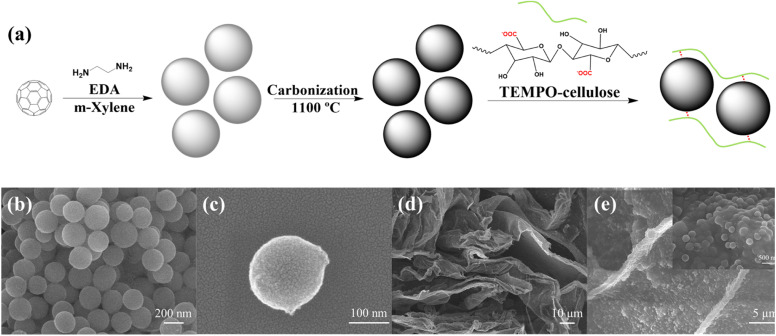
(a) Schematic illustration of the preparation process of C_60_-based carbonized spheres (CS)/TEMPO-cellulose aqueous solution. The SEM images of (b) CS aggregates and (c) single CS. (d) SEM image of a sheet-like structure of freeze-dried TCCS films. (e) SEM images showing CS dispersed in the TEMPO-cellulose polymer network.

As illustrated in Fig. 1(a), the C_60_-based nanospheres were obtained by crosslinking C_60_ and EDA. Therefore, the size of the nanospheres could be adjusted by controlling the molar ratio of C_60_ to EDA. As summarized in Fig. S1,† the size of C_60_-based nanospheres increased with increasing the C_60_ concentration in *m*-xylene from 0.25 to 2 mg mL^−1^. However, the nanospheres at 0.25 mg mL^−1^ exhibited a wide dispersion in size, ranging from 140 to 240 nm, while those at 2 mg mL^−1^ were distributed in the range of 340 to 400 nm, which did not meet the requirement for small particles to facilitate ion transfer. Hence, nanospheres prepared with 0.50 mg mL^−1^ of C_60_ in *m*-xylene were chosen to finish the following investigation as a relatively small and concentrated distributed size, mainly from 160 to 240 nm.

### Dispersion of carbonized spheres

The resultant C_60_-based nanospheres were carbonized at 1100 °C to obtain more hierarchically porous hollow CS. Similar to the original C_60_-based nanospheres, the CS owned a uniform spherical morphology with a diameter of around 200 nm (Fig. 1(b and c)). As previously reported, the resulting CS had an excellent large surface area (1439.3 m^2^ g^−1^) and large pore volume (1.346 cm^3^ g^−1^).^32^ Due to the lack of hydrophilic groups on the CS surface, CS could not be directly dispersed in water. To address this problem, we added CS to the TC aqueous solution. Based on the electrostatic absorption mechanism, when the neutral CS comes into contact with TC, positive charges are induced on its surface and interact with the negatively charged carboxylate groups of TC. The electrostatic interaction between TC and CS efficiently prevented the aggregation of CS, resulting in a stable and homogeneous dispersion of CS in the mixed solution.^33,34^ As shown in Fig. S2,† no CS precipitation was observed in the mixed solution after 24 h of storage at room temperature.

To further study the dispersion state of CS in a TC aqueous solution, the TC and TCCS aqueous solutions were freeze-dried and observed by SEM. As shown in Fig. 1(d and e) and S3,† both freeze-dried TC and TCCS possessed nanoporous network structures that can provide wide ion transport pathways. The CS was homogeneously dispersed in the network sheet due to its strong interaction with TC chains. Thus, CS could act as an additive to increase the mechanical strength and provide mesoporous pores for storing large amounts of charge because it facilitates the diffusion of electrolyte ions through the nanospaces.^32^

The CS carbonized at high temperatures always exhibits a graphitic structure and improved conductivity.^35,36^ To rule out the effect of electron transfer on supercapacitor performances, we measured the resistivity of the freeze-dried TCCS film by applying a voltage of −1 to 1 V. As shown in Fig. S4,† the TCCS had a large resistivity of 1.87 × 10^7^ Ω cm, indicating that the TCCS network is an insulator. Due to the insulating properties of the TCCS, the PVA-TCCS hydrogel electrolyte effectively suppressed electron transfer between the two electrodes, while free ion transfer was allowed. Thus, the risk of short circuits could be significantly reduced.

The thermal stability of TCCS was investigated by TGA and DTG. The freeze-dried TC and TCCS were heated from 20 to 1000 °C at a rate of 10 °C min^−1^. The TGA and DTG curves and the corresponding data are shown in Fig. S5 and Table S1,† respectively. The decomposition temperature (10% weight loss) of TCCS (247 °C) was improved compared to TC (237 °C). Besides, TC showed a higher decomposition rate at 263.7 °C than that of TCCS at 261.4 °C (Fig. S5†). The relatively good thermostability of TCCS could be attributed to the strong interaction between CS and TC.

The TCCS after TGA measurement was further observed by SEM. As shown in Fig. S6,† the network structure was maintained even when heated to such high temperatures. A large amount of CS was widely dispersed in the TC-based carbonized polymer matrix. The results repeatedly proved the stable interaction between TC and CS.

### Preparation of hydrogels

**Fig. 2:**
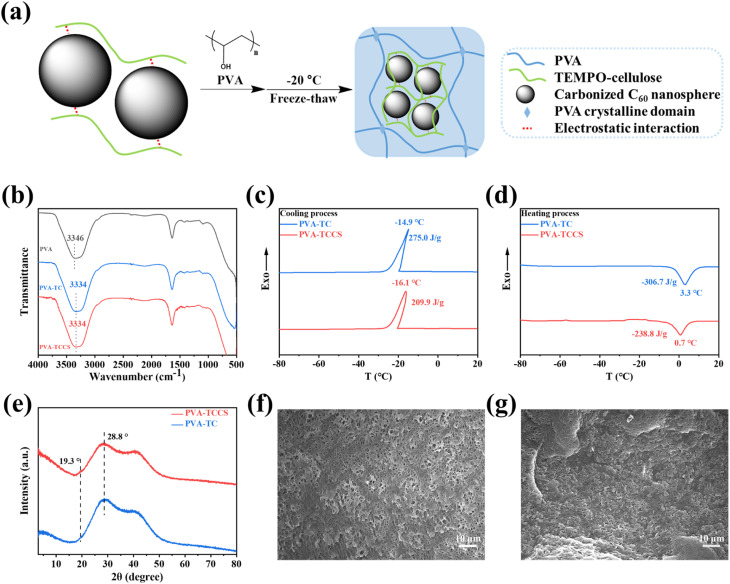
(a) Schematic illustration of the fabrication process of PVA-TCCS hydrogels. (b) FTIR spectra of PVA hydrogel, PVA-TC hydrogel, and PVA-TCCS hydrogel. DSC curves of PVA-TC hydrogel and PVA-TCCS hydrogel during (c) the cooling process and (d) the heating process. (e) XRD patterns of PVA-TC hydrogel and PVA-TCCS hydrogel. SEM images of the freeze-dried (f) PVA-TC hydrogel and (g) PVA-TCCS hydrogel.

Since TCCS aqueous solution cannot form a hydrogel by freeze-thawing process, PVA was further introduced into the TCCS aqueous solution system to obtain hydrogel-based electrolytes with flexibility and good mechanical properties. As shown in Fig. 2(a), PVA was dissolved in the homogeneous TCCS solution with stirring at 95 °C for 1 h. The hydrogels were then obtained by freeze-thawing the mixed solution. The strong electrostatic interaction between the carboxylate groups of TC and CS allowed CS to be stably dispersed in an aqueous solution during stirring. It is worth noting that PVA chains tend to form hydrogen bonds with TC chains rather than PVA chains. Therefore, the PVA-based hydrogel structure was reconditioned by TC to enhance its mechanical function.^37,38^ For the same reason, CS could not be dispersed in a PVA/TC aqueous solution because the carboxylate groups were occupied by PVA forming hydrogen bonds. The FTIR results also confirmed the interaction between PVA and TC. In Fig. 2(b), the peak of the hydroxyl group of the PVA hydrogel was 3346 cm^−1^. However, in the PVA-TC and PVA-TCCS hydrogels, the hydroxyl group peak significantly red-shifted to 3334 cm^−1^, indicating the formation of hydrogen bonds between PVA and TC.

DSC curves of the obtained hydrogels are displayed in Fig. 2(c and d). During the cooling process, the crystallization peaks of PVA-TC and PVA-TCCS occurred at −14.9 °C and −16.1 °C, respectively, which is attributed to the freezing point of free water in the hydrogels. Although there was a slight difference in the crystallization temperatures of PVA-TC and PVA-TCCS, the crystallization enthalpy of PVA-TCCS (209.9 J g^−1^) was much lower than that of PVA-TC (275.0 J g^−1^), suggesting that the CS dispersed in the hydrogel may inhibit the crystallization of the free water. A similar tendency was observed in the heating process. PVA-TCCS had a broad melting peak at 0.7 °C and a melting enthalpy of −238.8 J g^−1^, while PVA-TC showed a higher melting point (3.3 °C) and a higher melting enthalpy (−306.7 J g^−1^).

To investigate the crystallization of PVA in the hydrogels, XRD patterns of PVA-TC and PVA-TCCS were recorded. As shown in Fig. 2(e), a broad peak with strong intensity appeared at 28.8° for both PVA-TC and PVA-TCCS hydrogels, which is the result of the dense formation of physical crosslinks based on hydrogen bonds due to the repeated freeze-thaw cycles.^39^ Compared to PVA-TC, PVA-TCCS exhibited a weak peak at 19.3° corresponding to the (101) plane of PVA. Since the carboxylate groups of TC interacted with CS, PVA chains tended to form crystals with PVA chains.^40,41^

The morphologies of the PVA-TC and PVA-TCCS hydrogels were observed by SEM. PVA-TC (Fig. 2(f)) displayed a porous network structure with a very large pore size compared to PVA-TCCS (Fig. 2(g)). Because the CS of PVA-TCCS was wrapped in the polymer chains, the dispersion state could not be observed. However, the physical crosslinking point, CS, greatly increased the degree of crosslinking of the polymer chains, and PVA-TCCS showed a much denser polymer network structure.

### Mechanical properties

**Fig. 3:**
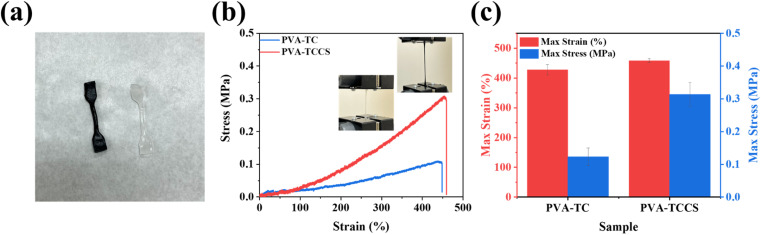
(a) Photo of the PVA-TC hydrogel (right) and PVA-TCCS hydrogel (left) samples for stretching tests. (b) Strain–stress curves of PVA-TC and PVA-TCCS hydrogels. (c) The max strain and max stress values obtained from the strain–stress curves.

As explained above, the strong electrostatic interactions between TC and CS help to build a uniform polymer network. Thus, CS could be an additive that improves the mechanical strength of the hydrogels. To investigate the effect of CS, strain–stress curves of PVA-TC and PVA-TCCS hydrogels were measured. All samples were cut into a dumbbell form of the same width, thickness, and length before measurement (Fig. 3(a)). As shown in Fig. 3(b) and S7,† the introduction of CS improved the maximum stress, while maintaining the maximum strain. As summarized in Fig. 3(c), the addition of CS improved the maximum strain from 427.67% to 458.33% and significantly increased the maximum stress from 0.12 to 0.31 MPa. The superior mechanical performance of PVA-TCCS hydrogels could be attributed to the semi-rigid stiffness of CS. During the stretching process, the semi-rigid CS effectively interfered with crack propagation due to micro-deformation under stress.^31,42^ In addition, CS also acted as a physical crosslinking point, improving the toughness of the resultant hydrogels. Based on both of the above properties of CS, the mechanical properties of PVA-TCCS hydrogels were effectively improved compared to PVA-TC hydrogels.

### Performance of hydrogel-based supercapacitor

**Fig. 4:**
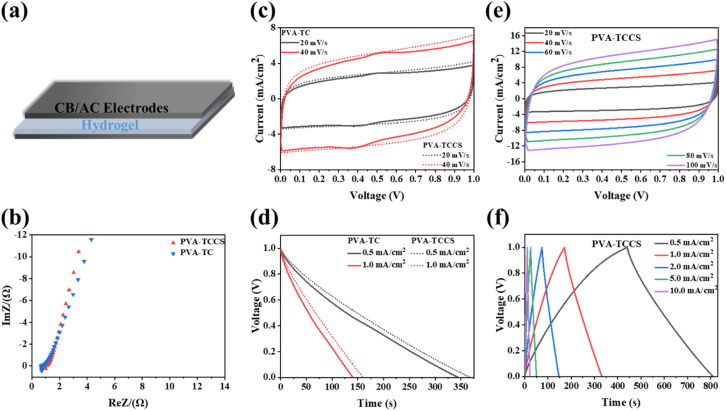
(a) Schematic representation of the assembled symmetric supercapacitor. (b) EIS curves of the PVA-TC hydrogel-based and PVA-TCCS hydrogel-based supercapacitors. (c) Comparison of CV curves at low scan rates (20 and 40 mV s^−1^) for the PVA-TC and PVA-TCCS hydrogel-based supercapacitors. (d) Comparison of discharge times of the PVA-TC and PVA-TCCS supercapacitors at 0.5 and 1 mA cm^−2^ (e) CV profiles of the PVA-TCCS system at different scan rates and (f) the corresponding CD profiles at different currents.

Before the fabrication of the supercapacitors, the ionic conductivities of the obtained hydrogel electrolytes were measured by EIS. As presented in Fig. S8 and Table S2,† the PVA-TCCS hydrogel showed a high ionic conductivity of 0.17 S cm^−1^, while the ionic conductivity of the PVA-TC hydrogel was 0.09 S cm^−1^. The higher ionic conductivity of the PVA-TCCS hydrogel is attributed to the mesoporous pores of CS, which can store a large number of charges. Due to the excellent high surface area, activated carbon is widely used in supercapacitor electrodes. The performance of the electrodes fabricated in this study was tested with a three-electrode setup: working electrode (activated carbon, conductive carbon black, and poly(vinylidene fluoride)), reference electrode (Ag/AgCl), and counter electrode (Pt). Electrochemical measurements (cyclic voltammetry (CV), galvanostatic charge/discharge (GCD), and electrochemical impedance spectroscopy (EIS)) were performed on the electrodes in an aqueous 1 M H_2_SO_4_ electrolyte. As shown in Fig. S9(a),† the Nyquist plots obtained from the EIS measurements slope ∼45° in the low-frequency region, suggesting a Warburg impedance resulting from oxygen in the electrode. The intercept on the *x*-axis at a high frequency represents the ohmic internal resistance, which was 1.1 Ω at this electrode. The rectangular shape of the CV curves shown in Fig. S9(b)† persisted at the scan rate from 20 to 100 mV s^−1^, indicating the fast diffusion of electrolyte ions due to the presence of porous channels. Since GCD profiles of the electrodes cannot be fully recorded by our electrochemical station because of the limitation of the longest charge–discharge time, the GCD curves of the electrodes were recorded at the current densities from 2 to 10 mA cm^−2^ (Fig. S9(c)†). As summarized in Fig. S9(d),† the resulting electrode had a high areal capacitance of 631.38 mF cm^−2^ at 2 mA cm^−2^ and reached 266.42 mF cm^−2^ at 10 mA cm^−2^. Therefore, the obtained electrodes showed a high and relatively stable performance in the liquid electrolyte, which can be further applied to the electrodes for hydrogel electrolyte-based supercapacitor devices. As shown in Fig. 4(a), a supercapacitor was fabricated by sandwiching a hydrogel-based electrolyte between two CB/AC electrodes of the same size. The electrochemical performances of the assembled hydrogel-based supercapacitor were comprehensively investigated in a two-electrode system. As shown in Fig. 4(b), the charge transport process of the assembled device was analyzed by EIS, and the results are plotted by Nyquist plots. The electrochemical impedance spectroscopy (EIS) plots of the two supercapacitors showed a nearly vertical tail at low frequencies, indicating good capacitance behavior.^43^ The intercepts of the real impedance axis at high frequencies were 0.68 Ω and 0.95 Ω, corresponding to the PVA-TC and PVA-TCCS hydrogel-based devices, respectively, which represent the internal resistance of the devices. The low resistance indicated that the electrodes adheres to the electrolyte and the interface contact resistance is reduced.^44^

The CV curves of the supercapacitors with different scanning speeds were measured. In the electrochemical window of 1 V, the CV curve of the supercapacitor with the PVA-TC hydrogel showed a symmetrical shape like a rectangle (Fig. S10(a)†). The electrochemical window was fixed at 1 V. The CV curves at different scanning speeds from 20 mV s^−1^ to 200 mV s^−1^ showed a rectangular shape at room temperatures, indicating that the device has a good capacitive performance.^45^ Compared to the PVA-TC-based device, the CV curve of the PVA-TCCS hydrogel-based device (Fig. 4(c and e)) showed a nearly perfect rectangle and a relatively larger window, indicating a faster and more efficient ion transfer.^43^ We also noticed a minor redox peak with insignificant intensity appeared in the CV curves of the PVA-TC hydrogel-based supercapacitor, whereas it disappeared in the CV curves of the PVA-TCCS hydrogel-based supercapacitor. This is due to the surface functionalities (oxygen) of the TEMPO-cellulose. The oxygen-based functional groups interacted with CS in the PVA-TCCS hydrogels, being electrochemically inactive. As a result, the PVA-TCCS system exhibited an electrical double-layer charge storage mechanism.

As shown in Fig. S10(b)† and 4(f), the GCD curves of both devices based on the PVA-TC and PVA-TCCS hydrogels at different current densities displayed a standard triangle and negligible voltage drop. It is noticeable that the GCD curves of both devices showed a nearly linear relationship between discharge/charge voltage and time, indicating an ideal electric double-layer capacitance behavior and fast charge–discharge capability.^1^ As shown in Fig. 4(d), the discharge time of the assembled supercapacitors suggested that the device based on the PVA-TCCS hydrogel has a longer discharge time and larger areal capacitance.

**Fig. 5:**
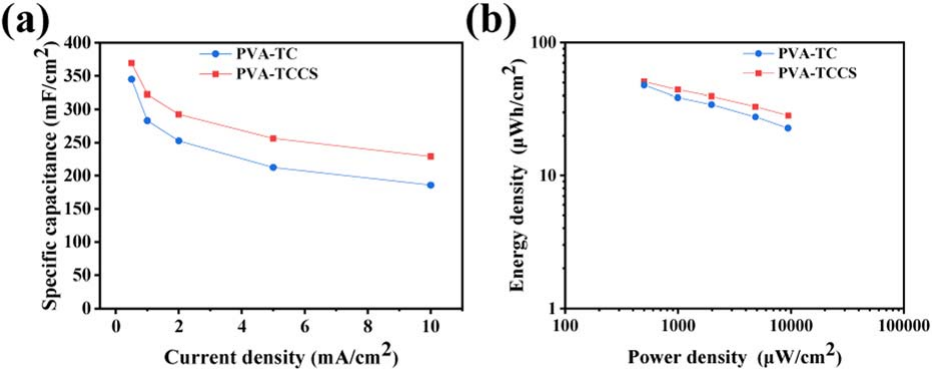
(a) Specific capacitance as a function of constant currents of the hydrogel-based supercapacitors. (b) Ragone plots of the hydrogel-based supercapacitors.

The specific areal capacitances calculated from the GCD curves at various current densities are shown in Fig. 5(a). The areal capacitances of the PVA-TC hydrogel-based device were 344.83 mF cm^−2^ at 0.5 mA cm^−2^ and decreased to 185.54 mF cm^−2^ at 10 mA cm^−2^, whereas the PVA-TCCS hydrogel-based device exhibited a larger capacitance throughout the operational current density. The PVA-TCCS hydrogel-based device had a high areal capacitance of 369.18 mF cm^−2^ at 0.5 mA cm^−2^, and the capacitance could reach 229.02 mF cm^−2^ at 10 mA cm^−2^. Therefore, the capacitance retention of the PVA-TCCS hydrogel-based device reached 62% at 10 mA cm^−2^, while that of the PVA-TC hydrogel-based device was only 53%. Due to the limitations in establishing the necessary interface with the electrode material, the hydrogel electrolyte-based solid-state supercapacitors have increased internal resistance and reduced charge storage properties, compared to similar liquid systems. Hence, the specific capacitance values obtained from the hydrogel-based supercapacitors were slightly lower than those obtained from the three-electrode setup with liquid electrolyte. Fig. 5(b) illustrates the energy density and power density of the assembled hydrogel-based supercapacitors, calculated from the GCD curves at different current densities. The maximum areal energy density of the PVA-TCCS hydrogel-based device reached 50.93 μW h cm^−2^ and the areal power density was 498.30 μW cm^−2^. As the areal power increased to 9450.47 μW cm^−2^, the areal energy density was still retained at 28.41 μW h cm^−2^. Similar to the capacitance performance, the PVA-TC hydrogel-based device showed a relatively lower energy density compared to the PVA-TCCS hydrogel-based device. Therefore, the introduction of mesoporous CS into the hydrogel-based electrolytes could effectively improve the capacitance performance by providing mesoporous pores to store a large amount of charge and promote ion diffusion in the nanospace.

## Conclusions

In this study, we introduced C_60_-based carbonized spheres (CS) into a PVA/TEMPO-cellulose-based hydrogel using the electrostatic interaction between the carboxylate groups of TC and CS. In addition, supercapacitors were assembled using the PVA-TC and PVA-TCCS hydrogels, prepared by a simple freeze-thaw process. Comprehensive characterization demonstrated the multiple effects of CS on the supercapacitor performance. First, the CS could act as physical crosslinking points, which help to improve the mechanical properties of the hydrogel by mitigating stress-induced crack propagation. Second, the nanospaces occupied by the CS significantly increased the ion transport efficiency. Therefore, this work provides new insights into the application of porous carbonized materials as solid electrolytes for the fabrication of energy storage devices.

## Author contributions

Han Jia: investigation and writing – original draft; Sabina Shahi: investigation; Lok Kumar Shrestha: conceptualization, investigation and writing – review & editing; Katsuhiko Ariga: conceptualization and writing – review & editing; Tsuyoshi Michinobu: supervision, funding acquisition and writing – review & editing.

## Conflicts of interest

There are no conflicts to declare.

## Supplementary Material

RA-013-D3RA03349J-s001
