# Supplementary material for: Improved supercapacitor performances by adding carbonized C60-based nanospheres to PVA/TEMPO-cellulose hydrogel-based electrolyte
Source: RSC Adv. 2023 Jul 18;13(31):21502–9. doi: 10.1039/d3ra03349j (PMC10352703; doi:10.1039/d3ra03349j)
Supplement: RA-013-D3RA03349J-s001 [file RA-013-D3RA03349J-s001.pdf]

Electronic Supplementary Material (ESI) for RSC Advances.
This journal is © The Royal Society of Chemistry 2023

Electronic Supplementary Information

Improved supercapacitor performances by adding carbonized C_{60}-based nanospheres to PVA/TEMPO-cellulose hydrogel-based electrolyte

Han Jia,[a] Sabina Shahi,[b] Lok Kumar Shrestha,[c,d,*] Katsuhiko Ariga,[c,e] and Tsuyoshi Michinobu[a,*]

[a] Department of Materials Science and Engineering, Tokyo Institute of Technology, 2-12-1 Ookayama, Meguro-ku, Tokyo 152-8552, Japan. E-mail: michinobu.t.aa@m.titech.ac.jp
[b] Central Department of Chemistry, Tribhuvan University, Kirtipur, Kathmandu 44613, Nepal
[c] Research Center for Materials Nanoarchitectonics (MANA), National Institute for Materials Science (NIMS), 1-1 Namiki, Tsukuba 305-0044, Japan. E-mail: SHRESTHA.Lokkumar@nims.go.jp
[d] Department of Materials Science, Faculty of Pure and Applied Sciences, University of Tsukuba, 1-1-1 Tennodai, Tsukuba 305-8573, Japan
[e] Department of Advanced Materials Science, Graduate School of Frontier Sciences, The University of Tokyo, 5-1-5 Kashiwanoha, Kashiwa 277-8561, Japan

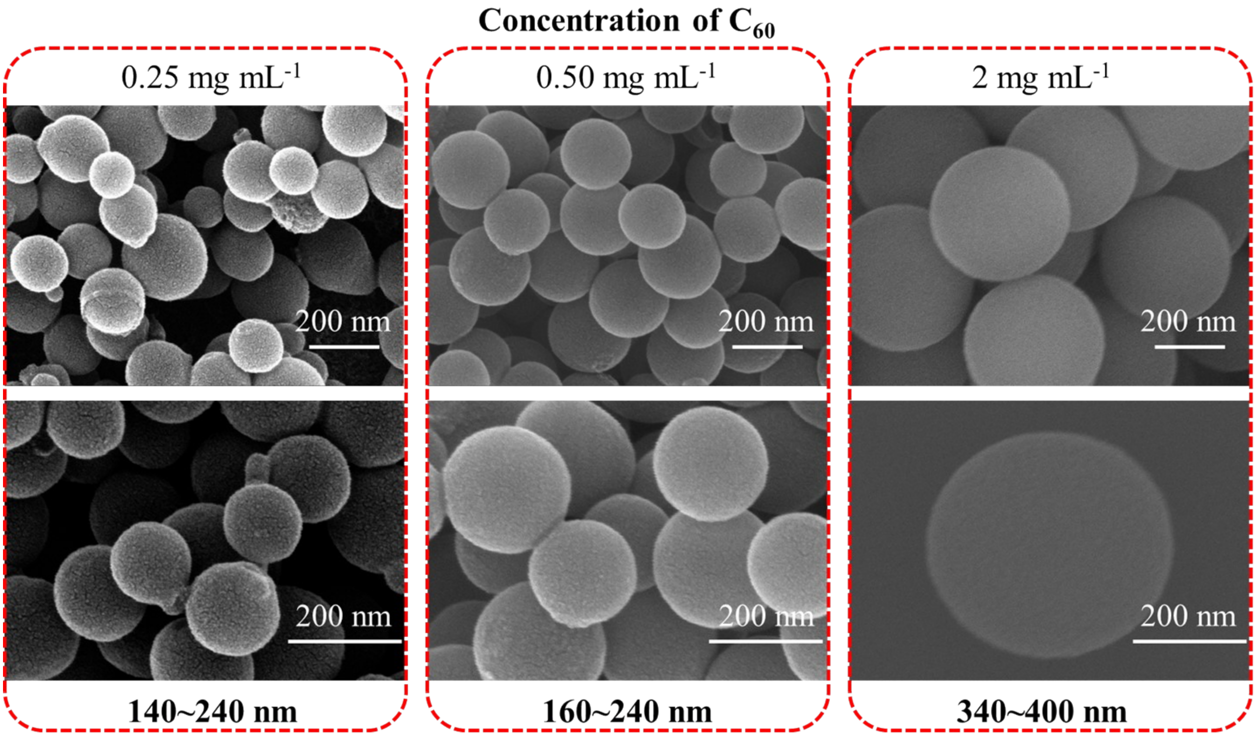

Fig. S1. SEM images of C_{60}-based nanospheres fabricated with different C_{60} concentrations.

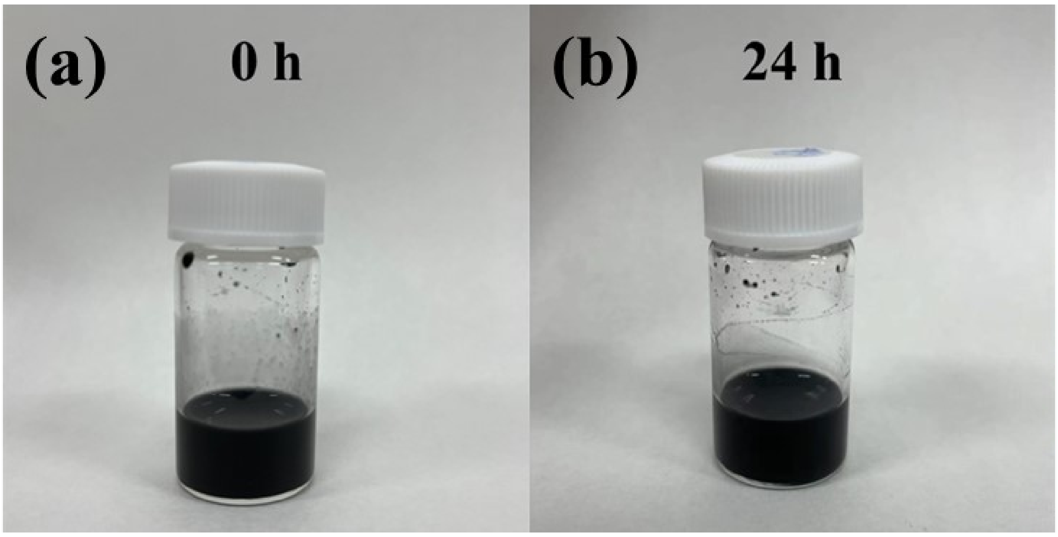

Fig. S2. Photos of TCCS aqueous suspension after (a) 0 h and (b) 24 h of storage.

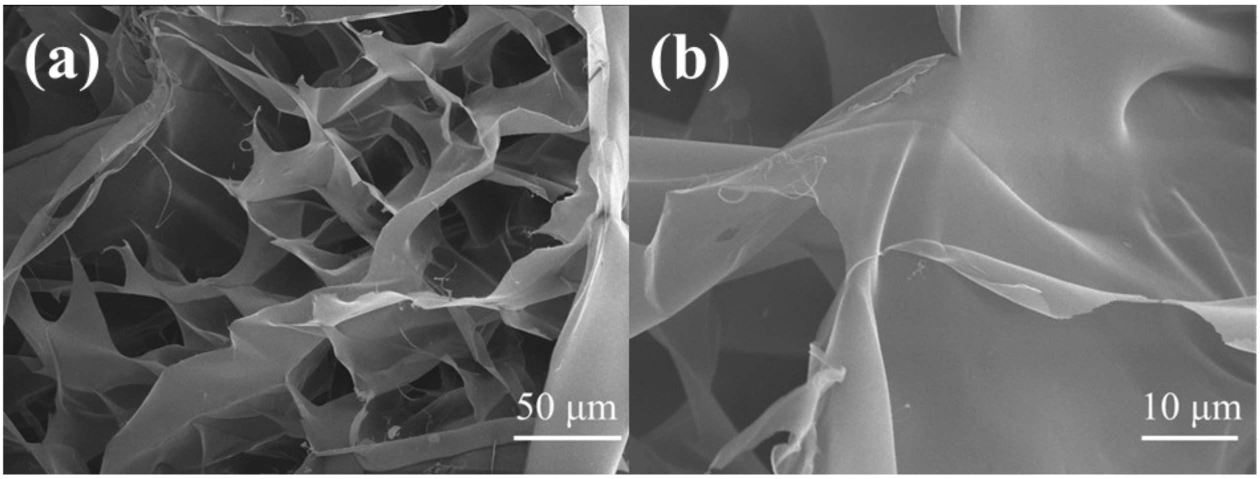

Fig. S3. SEM images of the porous structure of freeze-dried TC films. The image (b) is a magnified one of the image (a).

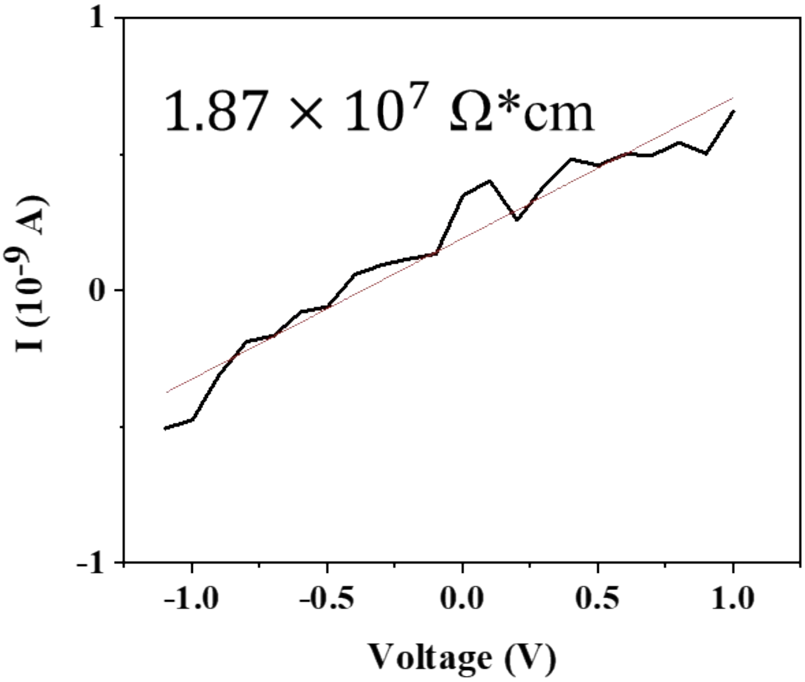

Fig. S4. The I-V curve of a freeze-dried TCCS film at a voltage of -1 V to 1 V. The red line is the fitted curve. The sample size was 0.8 cm × 0.1 cm × 0.03 cm (length × width × thickness).

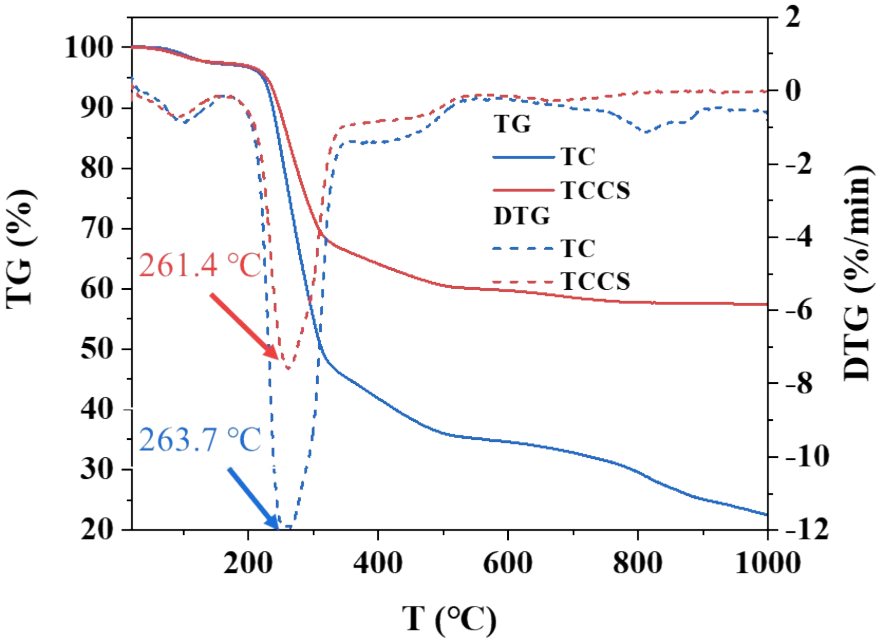

Fig. S5. TGA and DTG curves of freeze-dried TC and TCCS films from 20 to 1000 °C.

Table S1. Decomposition temperature (T_d) and residual amount after TGA measurements of the freeze-dried TC and TCCS films

Sample	T_d (90%) (°C)	Residual amount (%)
TC	237	22
TCCS	247	57

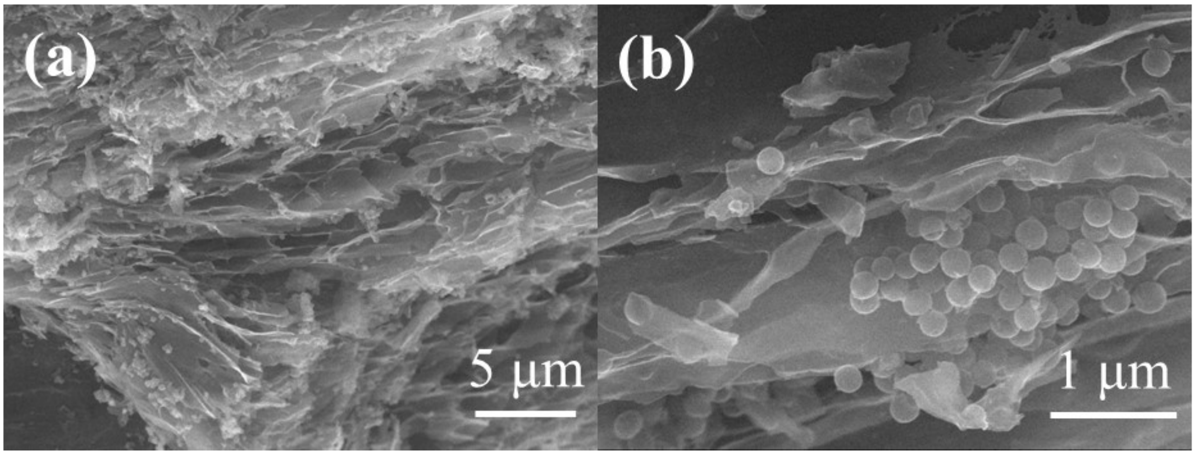

Fig. S6. SEM images of TCCS films after TGA measurements. The image (b) is a magnified one of the image (a).

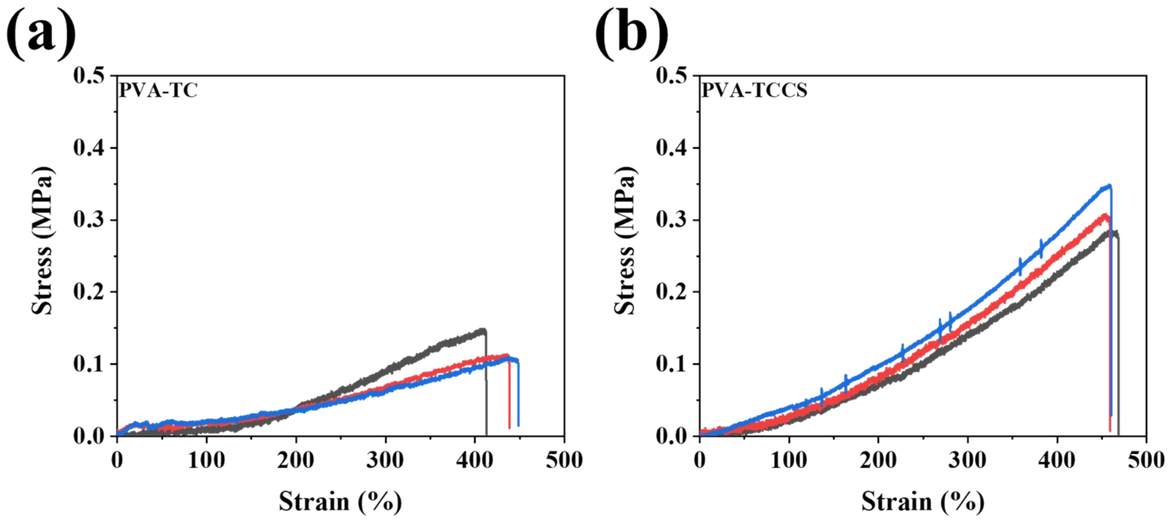

Fig. S7. Strain-stress curves of (a) PVA-TC and (b) PVA-TCCS hydrogels.

The hydrogels were sandwiched and sealed between two stainless steel discs for the measurement. The ionic conductivities (σ) of organogels were calculated according to the following equation:

$$\sigma = \frac{L}{RA} \qquad \text{(S1)}$$

where *L* represents the thickness of the hydrogel, *R* represents the impendence value obtained by the intercepts of EIS curves with the x-axis, and *A* represents the contact area of the organogel between two stainless steel electrodes.

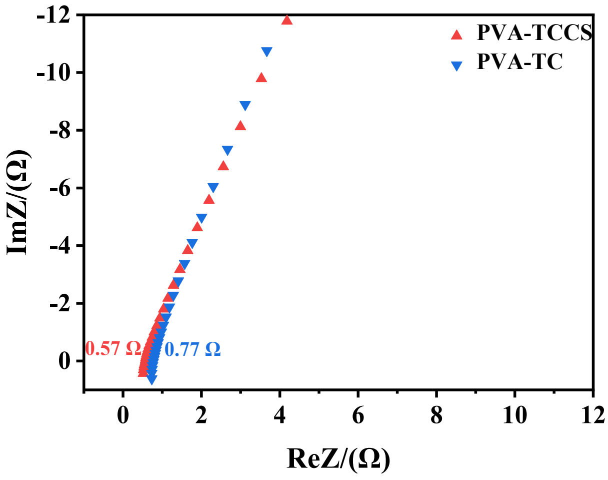

Fig. S8. The EIS plots of PVA-TC hydrogel and PVA-TCCS hydrogel after the solvent exchange with 1 M H_2SO_4 aqueous solution measured at a frequency range from 5×10^5 to 10^{-1} Hz and 10 mV voltage.

Table S2. Area, thickness, and ionic conductivity of the hydrogels measured by EIS.

Sample	Area (cm^2)	Thickness (cm)	Ionic conductivity ($S\ cm^{-1}$)
PVA-TC	1	0.10	0.17
PVA-TCCS	1	0.07	0.09

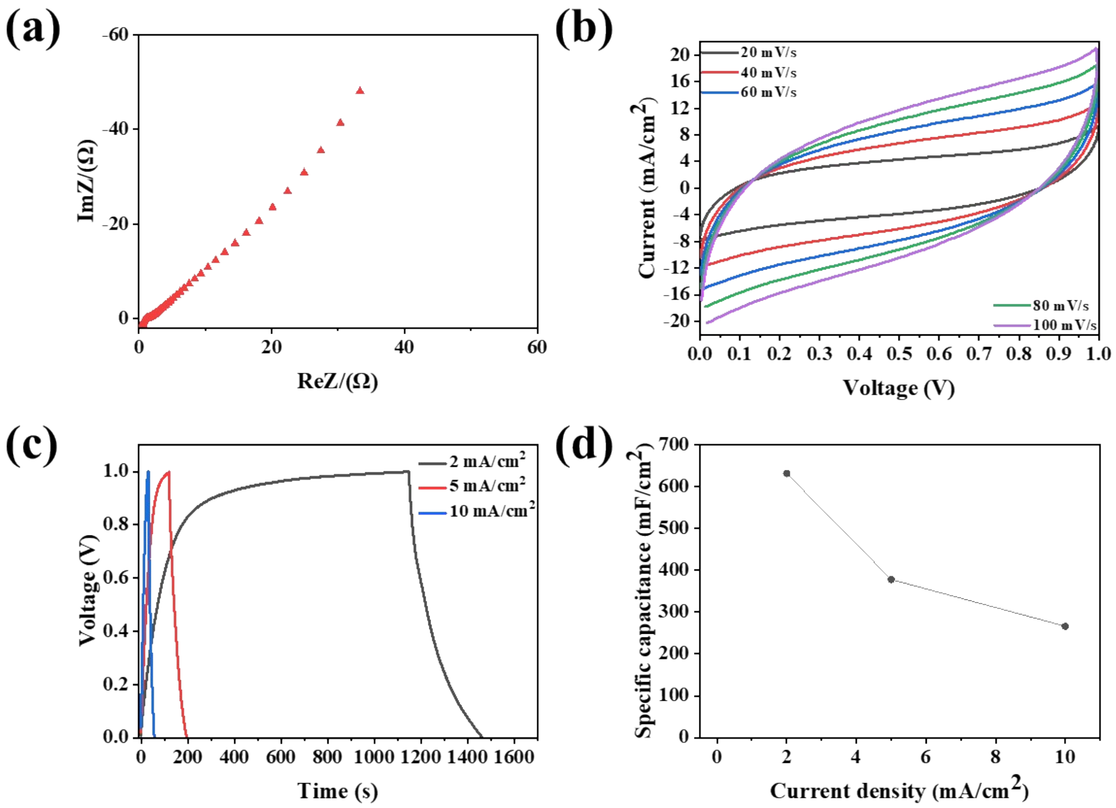

Fig. S9. (a) The Nyquist plots for the electrode. (b) CV curves vs. scan rate for the electrode. (c) GCD profiles vs. current density for the electrode. (d) Specific capacitance as a function of the current density of the electrode.

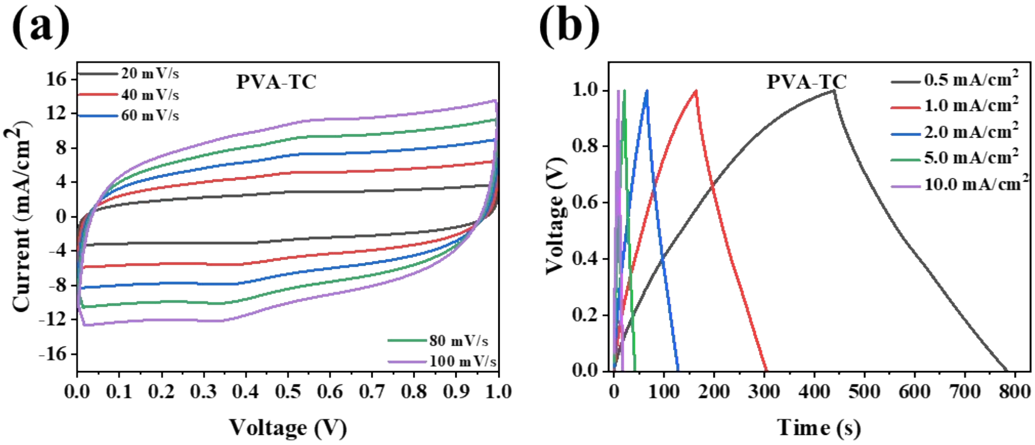

Fig. S10. (a) CV profiles of the PVA-TC system at different scan rates and (b) the corresponding CD profiles at different currents.
